# Metabolomics Diagnosis of COVID-19 from Exhaled Breath Condensate

**DOI:** 10.3390/metabo11120847

**Published:** 2021-12-06

**Authors:** Elettra Barberis, Elia Amede, Shahzaib Khoso, Luigi Castello, Pier Paolo Sainaghi, Mattia Bellan, Piero Emilio Balbo, Giuseppe Patti, Diego Brustia, Mara Giordano, Roberta Rolla, Annalisa Chiocchetti, Giorgia Romani, Marcello Manfredi, Rosanna Vaschetto

**Affiliations:** 1 Department of Translational Medicine, University of Piemonte Orientale, 28100 Novara, Italy; elettra.barberis@uniupo.it (E.B.); elia.amede@uniupo.it (E.A.); 20036967@studenti.uniupo.it (S.K.); luigi.castello@med.uniupo.it (L.C.); pierpaolo.sainaghi@med.uniupo.it (P.P.S.); mattia.bellan@med.uniupo.it (M.B.); giuseppe.patti@uniupo.it (G.P.); rosanna.vaschetto@med.uniupo.it (R.V.); 2 Center for Translational Research on Autoimmune and Allergic Diseases, University of Piemonte Orientale, 28100 Novara, Italy; annalisa.chiocchetti@med.uniupo.it; 3 Azienda Ospedaliero-Universitaria “Maggiore della Carità”, 28100 Novara, Italy; pieroemilio.balbo@maggioreosp.novara.it (P.E.B.); diego.brustia@maggioreosp.novara.it (D.B.); mara.giordano@med.uniupo.it (M.G.); roberta.rolla@med.uniupo.it (R.R.); 20025648@studenti.uniupo.it (G.R.); 4 Medicina Interna, Azienda Ospedaliera SS. Antonio e Biagio e Cesare Arrigo, 15121 Alessandria, Italy; 5 Department of Health Sciences, University of Piemonte Orientale, 28100 Novara, Italy

**Keywords:** breath analysis, metabolomics, COVID-19, GCxGC-MS, noninvasive analysis

## Abstract

Infection from severe acute respiratory syndrome coronavirus 2 (SARS-CoV-2) can lead to severe respiratory tract damage and acute lung injury. Therefore, it is crucial to study breath-associated biofluids not only to investigate the breath’s biochemical changes caused by SARS-CoV-2 infection, but also to discover potential biomarkers for the development of new diagnostic tools. In the present study, we performed an untargeted metabolomics approach using a bidimensional gas chromatography mass spectrometer (GCxGC-TOFMS) on exhaled breath condensate (EBC) from COVID-19 patients and negative healthy subjects to identify new potential biomarkers for the noninvasive diagnosis and monitoring of the COVID-19 disease. The EBC analysis was further performed in patients with acute or acute-on-chronic cardiopulmonary edema (CPE) to assess the reliability of the identified biomarkers. Our findings demonstrated that an abundance of EBC fatty acids can be used to discriminate COVID-19 patients and that they may have a protective effect, thus suggesting their potential use as a preventive strategy against the infection.

## 1. Introduction

Infection from severe acute respiratory syndrome coronavirus 2 (SARS-CoV-2) can lead to severe respiratory tract damage and acute lung injury. Autopsy reports on COVID-19 patients demonstrated diffuse alveolar damage and a wider spectrum of histological lesions affecting both the epithelial and vascular components of the lung [1]. Previous studies showed several biochemical changes in the breath in acute respiratory distress syndrome (ARDS) [2,3,4], and in viral (influenza A) and bacterial (*Streptococcus pyogenes*) infections [5]. Moreover, it is well-known that the oral–lung aspiration axis is a key factor that leads to many infectious diseases [6]. Therefore, it is critical to examine breath-associated biofluids not only to investigate breath-biochemical changes caused by SARS-CoV-2 infection, but also to discover potential biomarkers for the development of new diagnostic tests. Although the most reliable test to detect SARS-CoV-2 infection is real-time reverse-transcription polymerase chain reaction (RT-PCR) [7], new methods for the noninvasive diagnosis and monitoring of COVID-19 disease are needed. Computed tomography (CT) was proposed to evaluate the state of the lungs and virus progression, but radiologists concluded that, considering the pathognomonic imaging of COVID-19, CT cannot be use as a diagnostic tool.

As new diagnostic strategies are urgently needed to control the COVID-19 pandemic, the analysis of exhaled breath condensate (EBC) has been proposed for the identification of infected patients [8,9]. EBC, which is the liquid phase of the exhaled air sampled by cooling, represents a potential surrogate of the lung environment and of the lower respiratory tract of patients. It can be easily collected in a noninvasive manner, as EBC contains a variety of diluted, nonvolatile molecules (ranging from simple ions to DNA, leukotrienes, proteins, lipids, microbiota, etc.) and represents a promising source of biomarkers for both the diagnosis and monitoring of the disease. According to the first breath study conducted by Ruszkiewicz et al., patients with COVID-19 can be recognized from those with other conditions during initial healthcare contact [10]. In addition, Berna et al. found that volatile organic compound (VOC) analysis performed on EBC can be used to identify pediatric patients with SARS-CoV-2 infection [11], while Grassin-Delyle et al. were able to discriminate between COVID-19 acute respiratory distress syndrome (ARDS) and non-COVID-19 ARDS patients that were invasively mechanically ventilated [12]. EBC analysis is frequently used to investigate respiratory diseases, infections, obstructive disorders, and lung cancer [13,14,15].

Although these studies show promising results, it is not yet clear how EBC reflects the presence of SARS-CoV-2 and whether its composition might be associated with the virus. In addition, an extended and untargeted metabolomics characterization of EBC is still missing, since only VOC analysis or real-time, online, proton-transfer-reaction time-of-flight mass spectrometry (PTR-MS) technique have been used so far to analyze a limited number of molecules. The importance of extending the range of quantifiable molecules in total EBC is also supported by previous breath metabolite profiles obtained from other infections, including *Mycobacterium tuberculosis* (MT) and *Aspergillus* spp. [16], as well as pneumonia [17].

The aim of the present study was to perform an untargeted metabolomics approach using a bidimensional gas chromatography–mass spectrometer (GCxGC-TOFMS) on EBC from COVID-19 patients and negative healthy controls to identify new potential biomarkers for noninvasive diagnosis, monitoring, and clinical outcome prediction of COVID-19 pneumonia. The EBC analysis was further performed on patients with acute or acute-on-chronic cardiopulmonary edema (CPE) to determine the reliability of identified biomarkers, while healthy ex-COVID-19 subjects, negative for SARS-CoV-2 at the time of EBC collection, were analyzed to explore whether past infection with SARS-CoV-2 resulted in long-term changes in breath biochemistry.

## 2. Results

### 2.1. EBC Collection and Clinical Patients’ Characteristics

**Figure 1:**
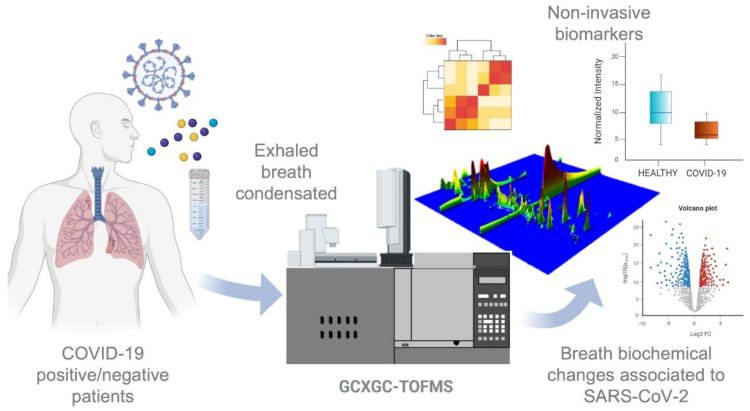
Experimental design of the study: exhaled breath condensate molecules were analyzed using untargeted metabolomics. Statistical analysis performed on quantified molecules was used to identify potential biomarkers and breath-biochemical changes associated with SARS-CoV-2 infection.

An untargeted metabolomics analysis was performed on exhaled breath condensate from COVID-19 patients, healthy volunteers, and CPE patients in order to investigate breath-biochemical alterations associated with viral infection and to identify new potential biomarkers (Figure 1). Table 1 summarizes the clinical features and demographics.

The patients’ median EBC volume collected was 800 (500–1100) μL, 825 (500–1050) μL, and 1130 (300–1200) μL for COVID-19, healthy volunteers, and CPE patients, respectively. The median age was 54 (44–64) years, 39 years (29–49), and 70 (66–76) years in COVID-19 patients, healthy volunteers, and CPE patients, respectively. For COVID-19 patients, the median ratio of partial pressure of oxygen to fraction of inspired oxygen (PaO_2_/FiO_2_) was 277 (241–338) mmHg with a median respiratory rate of 16 (15–18) breaths/min. A total of 36% of the COVID-19 patients presented an American Thoracic Society (ATS) [18] score that qualified pneumonia as severe, while all patients required oxygen support with nasal cannula or Venturi mask during hospitalization. The median hospital length of stay was 7 (5–10) days.

### 2.2. EBC Metabolome Is Influenced by SARS-CoV-2 Infection

**Figure 2:**
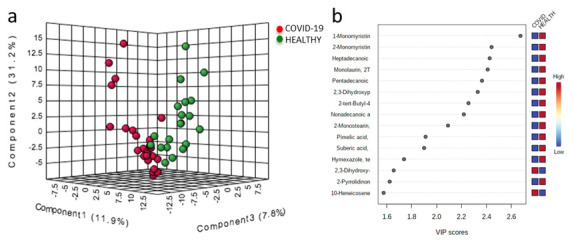
Partial least squares discriminant analysis (PLS-DA) of EBC metabolome from COVID-19 (red dots) and healthy subjects (green dots). The two groups are well-separated (**a**). Important features identified by PLS-DA (**b**): colored boxes indicate the most predictive or discriminative features in each group (red, high; blue, low).

From EBC analysis, 322 small molecules and some potential biomarkers were quantified, along with a unique signature associated with the SARS-CoV-2 infection. We first investigated the differences between COVID-19 patients and healthy subjects. As reported in the 3D score plot in Figure 2a, partial least squares discriminant analysis (PLS-DA) clearly shows the presence of a metabolomics profile associated with the infection. The most predictive or discriminative features that are potentially useful in helping sample classification were also determined through the variable importance in projection (VIP) score. The VIP score summarizes the most prominent molecules responsible for the reported phenotypic variances in COVID-19 EBC (Figure 2b).

### 2.3. Potential EBC Biomarkers of SARS-CoV-2 Infection

**Figure 3:**
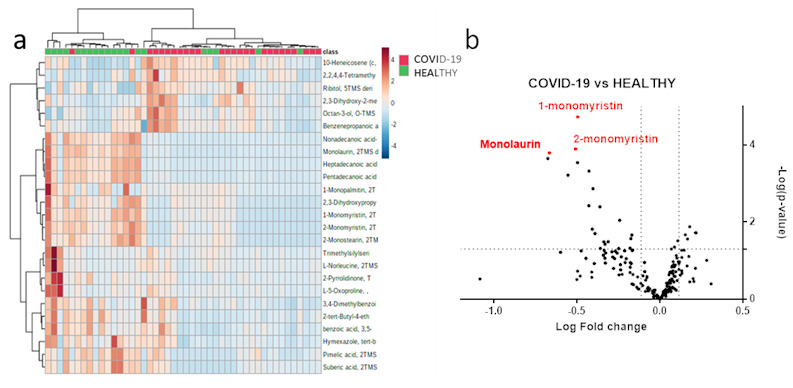
EBC-modulated metabolites in the comparison between healthy subjects versus SARS-CoV-2 patients. Hierarchical clustering heatmap of molecules in COVID-19 patients (red) and healthy subjects (green) (**a**). Volcano plot reporting 26 regulated small molecules with a *p*-value less than 0.05 and a fold change greater than 1.3 (**b**).

We then performed univariate statistical analysis on small molecule abundances. A total of twenty-six metabolites (Supplementary Table S1) were differentially expressed in EBC samples (*p*-value < 0.05 and fold change >1.3), as reported in the volcano plot in Figure 3b. Additionally, we performed hierarchical clustering heat-map analysis to better visualize the abundance of regulated metabolites and EBC sample grouping (Figure 3a).

**Figure 4:**
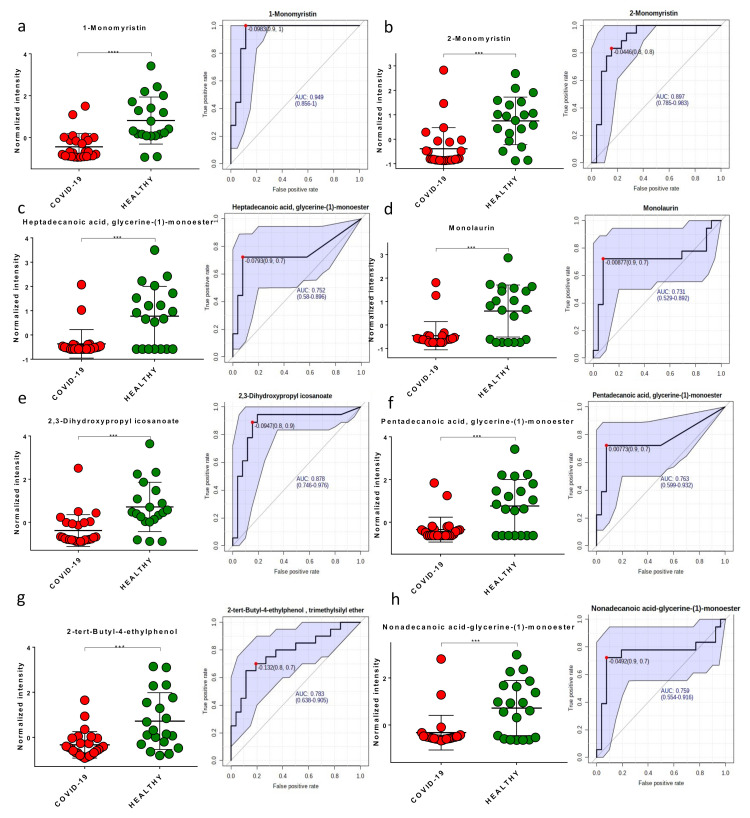
Boxplots and ROC curves for the best potential biomarkers identified with metabolomics analysis (red dots: COVID-19 patients, green dots: healthy subjects). 1-monomyristin (**a**); 2-monomyristin (**b**); heptadecanoic acid, glycerine-(1)-monoester (**c**); monolaurin (**d**); 2,3-dihydroxypropylicosanoate (**e**); pentadecanoic acid, glycerine-(1)-monoester (**f**); 2-tert-butyl-4-ethylphenol (**g**); nonadecanoic acid, glycerine-(1)-monoester (**h**). ***, *p*-value < 0.001; ****, *p*-value < 0.0001.

**Figure 5:**
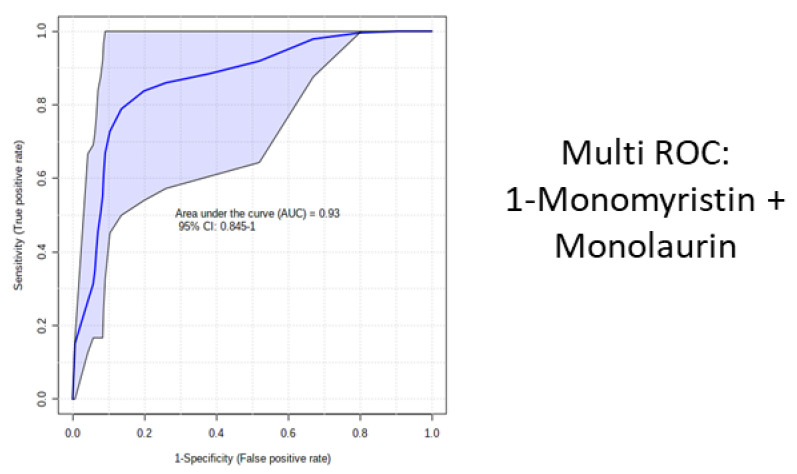
Combined ROC curve of the two best metabolites: 1-monomyristin and monolaurin.

Among the modulated molecules, we investigated potential biomarkers using boxplots and ROC curves. We discovered eight potential small molecules that appeared to be able to differentiate between COVID-19 patients and healthy subjects (Figure 4). The following molecules performed optimally in diagnostic tests: 1-monomyristin (AUC = 0.949); 2-monomyristin (AUC = 0.897); heptadecanoic acid, glycerine-(1)-monoester (AUC = 0.752); monolaurin (AUC = 0.731); 2,3-dihydroxypropylicosanoate (AUC = 0.878); pentadecanoic acid, glycerine-(1)-monoester (AUC = 0.763); 2-tertbutyl-4-ethylphenol (AUC = 0.783); nonadecanoic acid, glycerine-(1)-monoester (AUC = 0.759). The combined ROC curve of the two best molecules (1-monomyristin and monolaurin) revealed an AUC of 0.93 (Figure 5).

### 2.4. Validation of EBC Biomarkers with CPE and Machine Learning

We further investigated whether the identified biomarkers were able to distinguish COVID-19 patients from patients with CPE. The best previously selected biomarkers were not statistically different between CPE and COVID-19 patients; however, using the complete chemical fingerprints of small molecules and machine learning, we were able to discriminate the two groups of samples. We built a genetic algorithm machine learning model based on metabolomic data from 17 COVID-19 patients, 7 CPE subjects, and 16 healthy subjects that were randomly selected from our cohort and grouped based on SARS-CoV-2 positivity. The model was then externally validated on the remaining 17 subjects (COVID-19 *n* = 9; healthy *n* = 4; and CPE *n* = 4), reaching an average area under the curve of 0.98 (80 iterations) with a classification accuracy of almost always 100%. Interestingly, among the 20 most important features selected by the algorithm to perform the classification on, there were also best biomarkers obtained through monovariate analysis: monomyristin, monolaurin, heptadecanoic acid-glycerine-(1)-monoester, nonadecanoic acid-glycerine-(1)-monoester, pentadecanoic acid-glycerine-(1)-monoester, dihydroxypropyl icosanoate, 2-tert-butyl-4-ethylphenol, and monostearin. These results suggest that this approach can be used to discriminate COVID-19-positive patients not only from healthy controls but also from other patients with respiratory diseases, such as CPE.

Additionally, since the healthy group included eight subjects who had been infected in the past by SARS-CoV-2, we explored whether the SARS-CoV-2 infection caused long-term changes in breath biochemistry. We found no significant differences in the levels of SARS-CoV-2-breath-associated molecules between healthy subjects who had contracted COVID-19 and healthy controls who were never infected. This could be explained by the fact that healthy subjects who contracted COVID-19 had mild or no symptoms, with limited or no involvement of the respiratory tract.

## 3. Discussion

Although the analysis of EBC has been already proposed for the identification of SARS-CoV-2-infected patients, the only few studies reported in the literature are limited to a small number of patients or to a specific subset, such as pediatric [9], to the analysis of the volatile molecules [10,11,12], or to used instruments with a limited range of analyzed molecules such as GC-IMS [11] or PTR-MS [12].

Our results support the ability of the EBC–metabolomic approach to deeply investigate COVID-19 disease. Although most of the identified compounds were found at higher concentrations in healthy subjects, the comparison of EBC molecule abundances shows the presence of 26 metabolites that are significantly different between healthy subjects and COVID-19 patients. Interestingly, eight out of twenty-six molecules were monoglycerides of fatty acids. As reported, it is well-known that medium-chain fatty acids (MCFAs), long-chain fatty acids (LCFAs), and monoglycerides of fatty acids have antiviral activity and provide protection against viruses [19]. Among the best eight biomarkers identified, it is worth noting the presence of three saturate monoacylglycerols (monopalmitin, monomyristin, and monolaurin), which are downregulated in COVID-19 patients. These lipids are well-known for their antiviral properties and the ability to prevent intestinal coronavirus infections [20]. Both monomyristin and monopalmitin dysregulation in EBC have already been associated with an increased risk of lung cancer or infection susceptibility [21,22], and a significant decrease in monolaurin levels can be found in the serum of patients shortly before developing COVID-19 [23].

According to our metabolomics results, higher levels of monolaurin were found in the EBC of healthy controls compared with COVID-19 patients. A study investigating methods to reduce African swine fever virus infectivity showed that the addition of medium-chain fatty acids and monoacylglycerol laurate, in water and in pig’s feed, resulted in a reduction in infectivity [24]. Furthermore, Lerner et al. evaluated the effect of MCFAs in pig’s feed to prevent the spread of porcine epidemic diarrhea virus (PEDV), and found that the addition of medium-chain fatty acids to feed could decrease the detection of PEDV in food, thus reiterating the antiviral activity of medium-chain fatty acids [25]. Other fatty acids and fatty alcohols with potential viricidal effects were also tested on respiratory syncytial virus (RSV) cell lines, showing that their addition in food could protect infants against infection [26]. Interestingly, research focusing on rat bronchoalveolar lavage fluid discovered that the antibacterial activity of the bronchial mucus was attributable to a higher presence of free fatty acids [27].

Finally, the levels of the dicarboxylic pimelic acid in COVID-19 patients were lower than in healthy subjects, while 2,3-Dihydroxy-2-methylpropanoic acid showed an opposite trend, being found at higher concentrations in the EBC from COVID-19 subjects (Supplementary Table S1). These molecules may be involved in metabolisms associated with SARS-CoV-2 infection and disease development. On the other hand, research performed on NMR spectra of EBC from cats affected by asthma demonstrated that levels of several carboxylic and dicarboxylic acids, such as pimelic acid and suberic acid, were able to be used to distinguish healthy cats from cats with asthma [28].

The validation of potential biomarkers was performed on patients with CPE, which is a potentially fatal cause of acute respiratory failure and is characterized by symptoms similar to COVID-19. Although the biomarkers identified in the comparison between COVID-19 and healthy subjects were not completely suitable for discriminating CPE from COVID-19, the use of machine learning allowed for the correct classification of patients based on EBC metabolome, suggesting that this approach can be used to discriminate COVID-19-positive patients not only from healthy subjects but also from other patients with pulmonary diseases such as CPE.

Compared to previous research performed on adults [10,12] and children [11], we did not identify the markers previously found to be associated with COVID-19, including acetone, 2-butanone, octanal, heptanal, and nonanal. This could be mainly due to: (i) the different sample preparation methods and, in particular, to the different derivatization processes that, in our case, focused on diverse classes of molecules, although a GCxGC-MS instrument was used; (ii) different instrumentation, as the other authors used GC-IMS and PTR-MS; (iii) different patients’ immune responses, which not only depend on the age of the subject, but also on the severity and the progression of the disease.

The last striking result we obtained from this study is that we did not identify long-term changes in breath biochemistry from ex-COVID-19 subjects who had mild or no symptoms. The levels of SARS-CoV-2-breath-associated molecules were no different between healthy subjects who had contracted COVID-19 and healthy subjects who were never infected.

In conclusion, EBC contains different diluted molecules that might represent a promising source of biomarkers of SARS-CoV-2 infection and shows potential for use for earlier therapeutic intervention. Although previous studies have shown that breath analysis could be used to detect SARS-CoV-2-positive subjects, the identified biomarkers were mainly VOCs, alkanes, and volatile molecules. Our data demonstrate that an abundance of fatty acids can be used to discriminate COVID-19 patients and that they might have a protective role, thereby suggesting their potential use as a preventive strategy against infection. Although this is the largest untargeted GCxGC-MS research study in the literature to date, there are some limitations: the average age of COVID-19 patients in our cohort was higher than that of the healthy population, although the CPE patients were more similar in age; based on sample preparation, our analysis mainly focused on a few classes of molecules; and a larger validation would be required to confirm our results.

## 4. Materials and Methods

### 4.1. Patients

The study was performed at the university hospital “Maggiore della Carità” in Novara, Italy, between May 2020 and May 2021, according to the principles outlined in the Declaration of Helsinki. The protocol was approved by the Institutional Review Board (Comitato Etico Interaziendale Novara, protocol No. CE 116/20) and written informed consent was obtained from all subjects according to the Italian regulations. We enrolled the 26 patients, named COVID-19 group, with the following inclusion criteria: (1) SARS-CoV-2 positivity detected via reverse transcription PCR (RT-PCR) from nasopharyngeal swab; (2) bilateral COVID-19 pneumonia; (3) age ≥18 years; (4) respiratory distress treated with low-flow oxygen therapy; (5) ability to cooperate and breath trough the mouthpiece of the condenser (Turbo DECCS System, Medivac, Parma, Italy). A cohort of 20 healthy volunteers, named the healthy volunteer group, with negative SARS-CoV-2 reverse-transcription PCR (RT-PCR) from nasopharyngeal swab, was considered the control group. Furthermore, 11 patients with negative SARS-CoV-2 reverse-transcription PCR (RT-PCR) from nasopharyngeal swab, but were hospitalized with dyspneal symptoms due to acute or acute-on-chronic cardiopulmonary edema (CPE), were enrolled as a validation cohort. The sample size was calculated based on previous experiments on EBC from other laboratories; these analyses provided useful data on which to base an estimation of the variability.

### 4.2. Measurements

Demographic characteristics including height and weight, blood sample exams (white blood cell count, lymphocytes count, lactate dehydrogenase, D-dimer, ferritin, platelet count) and American Thoracic Society score [18] performed at hospital entrance, partial pressure of oxygen to fraction of inspired oxygen ratio (PaO_2_/FiO_2_) and respiratory rate performed on the day of EBC collection, volume of EBC collected, and coexisting comorbidities were recorded. Furthermore, hospital length of stay was registered.

### 4.3. EBC Collection

Patients were instructed to breathe through the mouthpiece. Once Turbo Deccs System (Medivac, Parma, Italy) was cooled at −4 °C, EBC was collected for 10 min. Samples were aliquoted and stored at −80 °C before analysis through bidimensional gas chromatography mass spectrometer.

### 4.4. Materials and Reagents

For the sample preparation, LC-MS-grade solvents and reagents were used. Methoxyamine, N,O-Bis(trimethylsilyl)trifluoroacetamide (BSTFA), tridecanoic acid, and hexadecane were purchased from Merck (Darmstad, Germany); water, acetonitrile, and pyridine were obtained from VWR (Milano, Italy); isopropanol (IPA) was from Scharlab (Barcelona, Spain).

### 4.5. Sample Preparation

A mixture of ACN/IPA/water (3:3:2) solution (1 mL) with tridecanoic acid as internal standard (1 ppm) was added to 500 µL of EBC and then vortexed. The sample was centrifuged at room temperature at 14,500× *g* for 15 min. The supernatant was dried using a speed-vacuum system. The derivatization was performed using methoximation (20 µL of methoxamine, 80 °C, 20 min) and BSTFA (50 µL, 80 °C for 20 min). Finally, an internal standard (5.55 µL of hexadecane) was added before the analysis.

### 4.6. GCxGC-TOFMS Analysis

The analyses of the samples were performed using a GCXGC-TOFMS with a dual-stage, quad-jet modulator. The MS instrument was a LECO Pegasus BT 4D (Leco Corp., St. Josef, MI, USA). As a first-dimension column, we used a 30 m Rxi-5Sil MS (Restek Corp., Bellefonte, PA, USA) capillary column (internal diameter = 0.25 mm) with a stationary phase film thickness of 0.25 µm, while the second-dimension chromatographic column was a 2 m Rxi-17Sil MS (Restek Corp., Bellefonte, PA, USA) with an internal diameter of 0.25 mm and a film thickness of 0.25 µm. For the carrier gas, we used high-purity helium (99.9999%) at a flow rate of 1.4 mL/min. A sample of 1 µL was injected in splitless mode with the inlet at 250 °C. The temperature program was as follows: (a) initial temperature at 70 °C for 2 min; (b) ramped 6 °C/min up to 160 °C, 10 °C/min up to 240 °C, 20 °C/min to 300; (c) held at this value for 6 min. The secondary column was maintained at +5 °C relative to the GC oven temperature of the first column. The programming rate was the same for both columns. Electron impact ionization was applied at 70 eV. The ion source temperature was set at 250 °C, the mass range was 25 to 550 *m/z* with an extraction frequency of 32 kHz for the bidimensional and 30 kHz for monodimensional analysis. The acquisition rates were 200 spectra/s for 2D analysis. The modulation period for the bidimensional analysis was maintained at 4 s for the entire run. The modulator temperature offset was set at +15 °C relative to the secondary oven temperature, while the transfer line was set at 280 °C [29,30,31].

### 4.7. Statistical and Data Analysis

Patient data are expressed as medians and interquartile ranges, or absolute numbers and percentages. The chromatograms were acquired in total ion current (TIC) mode. Peaks with signal-to-noise (S/N) ratio lower than 500.0 were rejected. ChromaTOF version 5.31 was used for raw data processing. Mass spectral assignment was performed by matching with NIST MS Search 2.3 libraries and the FiehnLib. The raw files were aligned with Statistical Compare version 4.74. The statistical analysis, the PLS-DA, and all processing was performed with MATLAB R2017 (The MathWorks Inc., Natick, MA, USA) [32], Metaboanalyst software 5.0, and GraphPad v. 7.

### 4.8. Machine Learning Analysis

Statistical analysis was supported by machine learning algorithms. We randomly divided the samples into two cohorts composed of 40 (training) and 17 (validation) subjects. From the training cohort, we selected important metabolite features with an information gain ratio feature selection algorithm, and we employed a genetic algorithm as the classifier algorithm. In the genetic algorithm analysis, parameters were set to 150 iterations, 10 population size, 0.8 crossover, and 0.1 mutation. Algorithms were built using R package caret (version 4.6.14) with 3-fold cross-validation repeated 5 times, the entire framework was repeated 80 times, and an average of accuracies was calculated. These selected important features were used for the genetic algorithm analysis on the independent test cohort (17 subjects).

## Figures and Tables

**Table 1:** Clinical characteristics of healthy volunteers, patients with acute and acute-on-chronic cardiopulmonary edema (CPE), and patients with COVID-19 pneumonia. Data are presented as absolute numbers (percentage) or as median values (interquartile range).

	Healthy Volunteers *n* = 20	CPE *n* = 11	COVID-19 *n* = 26
Age, years	39 (29–49)	70 (66–76)	54 (48–64)
Sex	M: 8 (40%) F: 12 (60%)	M: 6 (55%) F: 5 (45%)	M: 14 (54%) F: 12 (46%)
Weight, kg	61 (57–66)	80 (76–86)	75 (70–87)
Height, cm	170 (163–181)	170 (165–178)	168 (162–175)
EBC Volume, μL	825 (500–1050)	1130 (300–1200)	800 (500–1110)
ATS score (severe pneumonia)	/	/	8
SpO_2_, %	/	97 (96–98)	96 (96–97)
FiO_2_, %	/	21 (21–21)	29 (21–50)
Respiratory rate, breath/min	/	15 (10–16)	16 (15–18)
PaO_2_/FiO_2_, mmHg	/	330 (244–401)	277 (241–338)
Comorbidities			
Smoker	0	7 (54%)	3 (11%)
Ischemic cardiomyopathy	0	5 (38%)	2 (8%)
Valvulopathy	0	4 (31%)	1 (4%)
Hypertension	0	7 (54%)	8 (31%)
Obesity	0	3 (23%)	3 (11%)
Diabetes	0	4 (31%)	3 (11%)
Chronic respiratory failure	1 (6%)	3 (23%)	2 (8%)
Chronic renal failure	0	2 (15%)	0
Lab results at hospital admission			
White blood cell count, ×10^3^/μL	/	9.45 (7.32–11.86)	6.795 (5.28–9.62)
Lymphocyte count, ×10^3^/μL	/	1.82 (1.01–3.26)	0.97 (0.61–1.3)
Lactate dehydrogenase, U/L	/	423 (369–549)	547 (409–759)
D-dimer, μgFEU/L	/	805 (350–5201)	703 (487–1197)
Platelet count, ×10^3^/μL	/	247 (191–306)	220 (181–275)
Ferritin, ng/mL	/	40 (18–312)	294 (156–757)
Length of hospital stay, days	/	11 (7–26)	7 (5–10)

## Data Availability

Not applicable.

## Supplementary Materials

The following are available online at https://www.mdpi.com/article/10.3390/metabo11120847/s1, Table S1: modulated molecules.
